# The *lim-7p::ced-1::GFP* transgene from the MD701 strain increases embryonic lethality in *C. elegans*

**DOI:** 10.17912/micropub.biology.001420

**Published:** 2025-01-07

**Authors:** Nina M. Zampetti, Kristen A. Quaglia, Lisa N. Petrella

**Affiliations:** 1 Department of Biological Sciences, Marquette University, Milwaukee, Wisconsin, United States

## Abstract

The *lim-7p::ced-1::GFP* transgene has been widely used for evaluating germline apoptosis in *C. elegans* . Here we observed an increase in embryonic lethality in the MD701 strain that contains the *lim-7p::ced-1::GFP* transgene and a strain that outcrossed the *lim-7p::ced-1::GFP* transgene into the N2 wild-type strain. While the outcrossed strain had a significantly lower level of embryonic lethality than MD701, it still showed significantly higher embryonic lethality than wild type. Our results suggest that the presence of the *lim-7p::ced-1::GFP* transgene significantly increases embryonic lethality, but there may also be a secondary mutation in the MD701 strain that further increases embryonic lethality.

**Figure 1. The lim-7p::ced-1::GFP transgene leads to embryonic lethality when homozygous:**
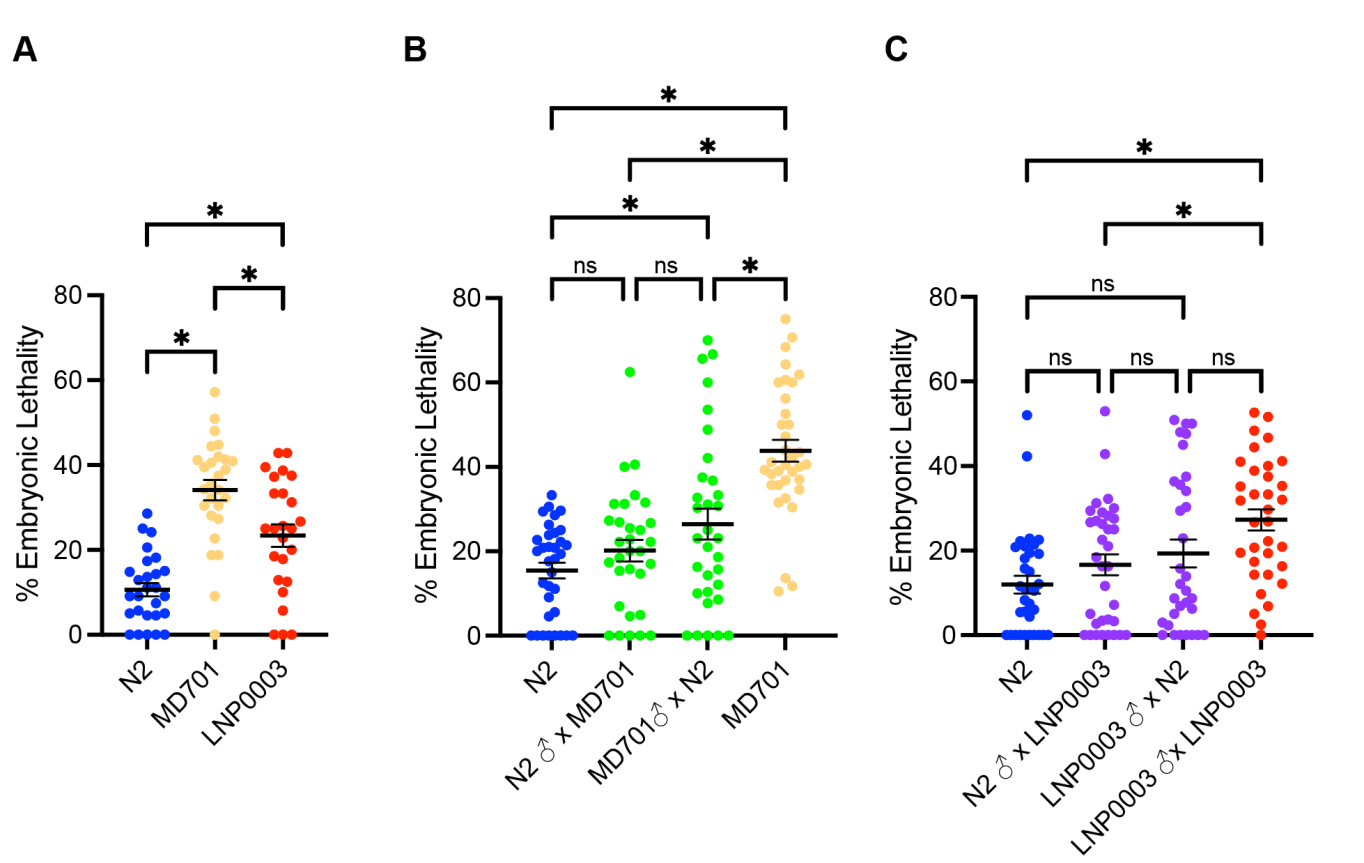
(A) Embryonic lethality measured at 20°C in the N2 wild-type strain, MD701 strain containing the homozygous multicopy *lim-7p::ced-1::GFP* transgene and the near isogenic backcrossed LNP0003 strain also containing the homozygous multicopy *lim-7p::ced-1::GFP* transgene. (B) Embryonic lethality measured in N2 and MD701 backgrounds compared to F1 cross progeny that are heterozygous for the multicopy *lim-7p::ced-1::GFP* transgene inherited either from the maternal or paternal parent. (C) Embryonic lethality measured in N2 and LNP0003 backgrounds compared to F1 cross progeny that are heterozygous for the multicopy *lim-7p::ced-1::GFP* transgene inherited either from the maternal or paternal parent. * significantly different *P-* value < 0.05 using one-way ANOVA with Tukey correction. Error bars indicate +/- SEM.

## Description

*Caenorhabditis elegans* is a commonly used model organism in the study of apoptosis. The MD701 strain is a reagent used to visualize germline apoptosis in live animals. MD701 contains the *bcIs39* transgene, which contains an integrated multicopy array of the *ced-1::GFP* transgene driven under the *lim-7* promoter (Zhou et al. 2001, Schumacher et al. 2005) . CED-1::GFP is thus expressed predominately in the somatic gonad sheath cells, which allows for visualization of apoptotic germ cells as they are engulfed (Zhou et al. 2001) . MD701 and other strains derived from it containing *bcIs39* are easy to maintain and have been used extensively across many studies (Salinas et al. 2006, Morrison et al. 2014; Manage et al. 2020; Polli et al. 2020; Fausett et al. 2021) .

While working on an embryo lethality assay for another project, we observed that MD701 showed an unusually high level of embryonic lethality. Upon full analysis we found the MD701 showed a significantly higher level of embryonic lethality than the N2 wild type strain ( Fig. 1A ). To determine if the embryonic lethality was due to a secondary mutation that had accumulated in the MD701 strain, we backcrossed the *lim-7p::ced-1::GFP* transgene into our N2 strain to create the near-isogenic LNP0003 strain. We found that the LNP0003 strain showed a level of embryonic lethality that was intermediate between the MD701 and N2 strains but was still significantly higher than N2 ( Fig. 1A ). Next, we created male lines of all three strains using heat shock and used these males to create cross progeny between the N2 wild type strain and each *lim-7p::ced-1::GFP* transgene containing strain. Crosses were set up in both directions so the F1 progeny where heterozygous for the *lim-7p::ced-1::GFP* multicopy transgene inherited either maternally or paternally. For the experiments where *lim-7p::ced-1::GFP* was inherited from the MD701 background there was a significant increase in embryonic lethality compared to N2 only when the *lim-7p::ced-1::GFP* was paternally inherited ( Fig. 1B ). However, this cross also showed a significantly lower level of embryonic lethality compared to MD701 . For experiments where *lim-7p::ced-1::GFP* was inherited from the LNP0003 background, neither cross showed an increase in embryonic lethality compared to N2 ( Fig. 1C ). Interestingly, when the *lim-7p::ced-1::GFP* was maternally inherited, the level of embryonic lethality was significantly lower than the LNP0003 background. Over all our data suggest that there is a significant increase in embryonic lethality with the *lim-7p::ced-1::GFP* multicopy transgene.

Our initial analysis of the level of embryonic lethality in N2 , MD701 , and LNP0003 suggests that the *lim-7p::ced-1::GFP* locus itself likely results in increased embryonic lethality. Previous work showed an increase in embryonic lethality in a strain containing the MD701 derived *lim-7p::ced-1::GFP* transgenes (Li et al. 2022) . However, in this work the strain being used also contained the *lag-2p::mCherryPH* transgene, which labels the distal tip cell (Pekar et al. 2017; Li et al. 2022; ). Since we see a similar increase in embryonic lethality in strains lacking the *lag-2p::mCherryPH* transgene it suggests that it is the *lim-7p::ced-1::GFP* transgene that is the causative allele. The embryonic lethality we saw with the LNP0003 strain was consistently significantly less than what we saw with the MD701 strain, which also suggest there may be a contributing secondary mutation that has arisen in the MD701 strain. Since it is not know where on chromosome V the *lim-7p::ced-1::GFP* insertion lies, there is not currently a clear candidate gene that could be a second site mutation linked to the transgene. Sequencing across the MD701 and LNP0003 lines looking for differential mutations present in MD701 and not LNP003 could help determine if there are candidate mutations for further investigation.

Interestingly, there was less/no increased embryonic lethality when the mother laying the embryo was heterozygous for the *lim-7p::ced-1::GFP* (Fig 1B-C). The most likely explanation of this is that homozygous expression of *lim-7p::ced-1::GFP* in embryos is directly causing the embryonic lethality. The *lim-7* promoter has been shown to be expressed in the embryo (Reece-Hoyes et al. 2007) . Therefore, even though GFP expression is not obviously present by eye in *lim-7p::ced-1::GFP* embryos, it is likely that the transgene is expressed in embryos and could cause embryonic lethality through expression of CED-1 in the wrong place/time. However, we cannot rule out that high levels of expression in the somatic gonad of mothers homozygous for the *lim-7p::ced-1::GFP* transgene is having an effect on embryonic lethality. Because many genes that have function in the somatic gonad also have roles in essential roles embryonic development (Hubbard and Greenstein 2000), it has been difficult to study how changes in somatic gonad function can directly or indirectly affect embryonic viability through changes in the germline or initial formation of embryos. But at least one work has shown that changes in the gonad sheath can result in increased embryonic lethality due to resulting changes in embryonic eggshells (Choi and Ambros 2019) . Whether this could be the case for embryos from animals expressing *lim-7p::ced-1::GFP* remains to be investigated. However, no matter the cause of the increase in embryonic lethality, labs choosing to use *lim-7p::ced-1::GFP* to analyze germline apoptosis need to be careful if they want to extend those analyses to oocyte or embryonic phenotypes when using MD701 and other strains containing *bcIs39* .

## Methods

***C. elegans* husbandry and maintenance**

All strains were maintained using standard methods (Brenner, 1974) on nematode growth media (NGM) plates. All strains were fed AMA1004 *E. coli* and kept at 20 ^o^ C. Strains utilized in this study were the wild type N2 ; MD701 *bcIs39 [lim-7p::ced-1::GFP; lin-15 (+)] V* and LNP0003 *petIR 3 (V; bcIs39 [lim-7p::ced-1GFP: lin-15 (+)] N2 > N2 ) ,* a near isogenic strain obtained from outcrossing the MD701 strain into the lab N2 strain 5 times. **The MD701 strain was provided by the CGC, which is funded by NIH Office of Research Infrastructure Programs (P40 OD010440).**

**Embryonic lethality assays**

To score embryo lethality, eight L4s from each strain were shifted to a new plate and incubated at 20°C for 24 hours. From that plate, seven young adults were cloned out onto individual thin lawn plates (50µL of lysogeny broth inoculated with AMA1004 spread on each NGM plate and allowed to grow for 24 hours before storing at 4°C). The young adults were incubated at 20°C for 6 hours and then removed from plates. The number and location of embryos was scored and recorded directly after young adults were removed. Plates were then incubated at 20°C for another 24 hours and then the number of hatched worms was counted and recorded. Scoring method involved stenciling a grid onto the back of the plate to use for reference to prevent against missing progeny or double counting. Raw data was recorded into Excel and statistics and graphs were done with Prism 10.0.3 (GraphPad Boston, MA).

**Crossing for heterozygous strain analysis**

To create males for crosses that would contain only the DNA from a given strain, four plates of 4-5 L4s were incubated for 6 hours at 30°C and then males were picked from the progeny and maintained as male/hermaphrodite strains on separate plates from hermaphrodites only.

To assess embryonic lethality in strains that were heterozygous for one genetic background with only one copy of the *ced-1::GFP* transgene we did the following: three days before the start of the embryonic lethality assay 10 male worms were put onto a plate with 2 hermaphrodites. To select F1 cross progeny for embryonic lethality assays we did the following using an SMZ1500 fluorescent stereoscope: For MD701 ♂ x N2 and LNP0003 ♂ x N2 crosses, L4 animals were cloned out from cross plates on Day 3. Then on Day 4 only adults with visible *ced-1::GFP* expression were cloned out to thin lawn plates for embryo laying. For N2 ♂ x MD701 and N2 ♂ x LNP0003 L4 animals were cloned out from cross plates on Day 3. Then on Day 4 only adults with dimmer *ced-1::GFP* expression were selected, by comparing to the plate of adult MD701 individuals for what constituted “bright” versus “dim” to ensure cross progeny were assayed instead of MD701 or LNP0003 self-progeny. Embryonic lethality assays were then performed as described above.

## Reagents

**Table:** 

Strain Name	Genotype	From
N2	Wild type	Susan Strome
MD701	*bcIs39 [lim-7p::ced-1::GFP; lin-15 (+)]V *	CGC
LNP0003	* petIR 3 (V; bcIs39 [lim-7p::ced-1GFP: lin-15 (+)] N2 > N2 ) *	This study
